# India Celebrates Its First National Physical Medicine and Rehabilitation Day (July 6, 2025): A Step Toward Disability Prevention

**DOI:** 10.7759/cureus.88165

**Published:** 2025-07-17

**Authors:** Raktim Swarnakar

**Affiliations:** 1 Physical Medicine and Rehabilitation, National Cancer Institute (NCI) Jhajjar Campus, All India Institute of Medical Sciences, New Delhi, IND

**Keywords:** awareness campaign, disability prevention, india, physical medicine and rehabilitation, universal health coverage (uhc)

## Abstract

India recently marked its first nationwide Physical Medicine and Rehabilitation (PMR) Day, highlighting the critical role of rehabilitation in preventing disability through early and proactive care. Centered around the theme of timely intervention and functional recovery, the initiative aimed to integrate PMR more effectively within the healthcare system. Events held across medical institutions showcased the growing recognition of rehabilitation as vital in managing chronic diseases, injuries, and age-related conditions. The observance also underscored the need for stronger policies, a skilled workforce, and accessible infrastructure to ensure equitable rehabilitation services. By promoting interdisciplinary collaboration and prioritizing community reintegration, this initiative represents a significant step toward a more inclusive, patient-centered healthcare model in India.

## Editorial

India observed its inaugural Physical Medicine and Rehabilitation (PMR) Day on July 6, 2025, marking a significant milestone in the country’s evolving approach to healthcare [1]. With the theme "Prevent Disability Before It Happens," the day was not just symbolic but a vital call to action, encouraging early intervention, functional restoration, and a holistic view of health [1]. Spearheaded by the Indian Association of Physical Medicine and Rehabilitation (IAPMR), this national observance-marked by the participation of various autonomous medical institutes, along with central, state, and private medical institutions-highlights the growing recognition of PMR as an essential component in promoting well-being, reducing disability, and improving quality of life across all age groups [1]. PMR, also known as physiatry, is a medical specialty that focuses on enhancing and restoring functional ability and quality of life to those with physical impairments or disabilities [2]. Unlike other specialties that focus primarily on curing disease, PMR emphasizes optimizing function and participation [2]. In a country like India, with a growing burden of non-communicable diseases (NCDs), trauma, aging populations, and postoperative functional limitations, the role of rehabilitation cannot be overstated.

According to the World Health Organization, over 2.4 billion people globally could benefit from rehabilitation, a number that is expected to rise due to the aging population and increasing prevalence of chronic diseases [3]. India, with its vast and diverse population, faces unique challenges: delayed referrals, limited access to rehabilitation services, low awareness among primary care physicians and the public, and a fragmented rehabilitation infrastructure [4]. Observing a national PMR Day is thus a strategic step toward raising awareness, encouraging policy-level discussions, and promoting an integrated model of care that includes timely rehabilitation services, such as community outreach initiatives and training programs, particularly at the primary healthcare (PHC) level, to maximize its impact. The timing of this observance is also crucial. India is currently transitioning toward Universal Health Coverage (UHC), and integrating rehabilitation into primary and secondary care levels is imperative [4]. Rehabilitation should no longer be perceived as an afterthought or luxury, but as a core health service. PMR bridges the gap between acute medical care and long-term recovery, making it indispensable in post-stroke care, spinal cord injury, traumatic brain injury, cancer survivorship, musculoskeletal conditions, and chronic pain management.

In rural and underserved areas, the absence of timely rehabilitation often results in avoidable disabilities [4]. Furthermore, the involvement of PMR professionals in acute care settings has been shown to enhance functional outcomes and promote a more seamless and integrated continuum of care [5]. India’s first PMR Day is also a step toward empowering the next generation of healthcare providers. Events organized on this day aimed to introduce them to the field's scope, patient impact, and interdisciplinary nature. Initiatives like this can inspire a stronger workforce in rehabilitation sciences, which is currently underrepresented [1]. Moreover, the celebration serves as an advocacy tool to urge policymakers to prioritize investments in rehabilitation infrastructure. This includes expanding PMR departments in tertiary hospitals, developing community-based rehabilitation models, integrating rehabilitation into policy, and funding research in neurorehabilitation, pain management, and assistive technology. The government’s support will be critical in ensuring that rehabilitation services are not just available but accessible and affordable to all.

This initiative also aligns closely with India’s broader push toward UHC. As the country reorients its healthcare priorities, rehabilitation must be viewed not as an optional service but as a core component of comprehensive care [4]. PMR plays a pivotal role in bridging the gap between acute medical intervention and long-term recovery [5]. It is indispensable in managing conditions such as stroke, spinal cord injury, traumatic brain injury, cancer survivorship, musculoskeletal disorders, and chronic pain [2,5]. However, these efforts often operate in silos. PMR Day offers a unifying platform that can tie these threads together, focusing attention on functional outcomes and long-term well-being. Importantly, the theme "Prevent Disability Before It Happens" reflects a shift from reactive to proactive healthcare [1]. This philosophy is gaining traction globally, with rehabilitation now recognized as a core part of universal health systems. India’s inclusive approach-emphasizing early intervention, system-wide integration, and capacity building-echoes this global shift, reaffirming its commitment to strengthening rehabilitation within UHC [4].

To sum up, the first national observance of PMR Day is more than just a ceremonial milestone. It is a strategic move toward mainstreaming rehabilitation in India’s healthcare discourse. By emphasizing early intervention, promoting awareness, and advocating for policy change, this initiative has the potential to transform the way India addresses disability. As the country moves forward, sustained efforts, intersectoral collaboration, and community engagement will be key to realizing the vision of a disability-free, functionally empowered India.
